# Targeting Genome Stability in Melanoma—A New Approach to an Old Field

**DOI:** 10.3390/ijms22073485

**Published:** 2021-03-28

**Authors:** Marta Osrodek, Michal Wozniak

**Affiliations:** Department of Molecular Biology of Cancer, Medical University of Lodz, 92-215 Lodz, Poland; marta.osrodek@stud.umed.lodz.pl

**Keywords:** melanoma, targeted therapy, MAPK inhibitors, immune checkpoint inhibitors, drug resistance, DNA damage, DNA repair

## Abstract

Despite recent groundbreaking advances in the treatment of cutaneous melanoma, it remains one of the most treatment-resistant malignancies. Due to resistance to conventional chemotherapy, the therapeutic focus has shifted away from aiming at melanoma genome stability in favor of molecularly targeted therapies. Inhibitors of the RAS/RAF/MEK/ERK (MAPK) pathway significantly slow disease progression. However, long-term clinical benefit is rare due to rapid development of drug resistance. In contrast, immune checkpoint inhibitors provide exceptionally durable responses, but only in a limited number of patients. It has been increasingly recognized that melanoma cells rely on efficient DNA repair for survival upon drug treatment, and that genome instability increases the efficacy of both MAPK inhibitors and immunotherapy. In this review, we discuss recent developments in the field of melanoma research which indicate that targeting genome stability of melanoma cells may serve as a powerful strategy to maximize the efficacy of currently available therapeutics.

## 1. Introduction

The genome of all living cells is constantly exposed to insults that generate DNA modifications, frequently causing DNA damage and affecting the ability of cells to survive and divide. DNA lesions include modified or mismatched bases, bulky DNA adducts, single-strand and double-strand breaks as well as crosslinks [1,2]. In the course of evolution, a large network of repair mechanisms has developed to repair all types of DNA damage: direct damage reversal [3], mismatch repair (MMR) [4], base excision repair (BER) [5], nucleotide excision repair (NER) [6], homologous repair (HR) [7], non-homologous end joining (NHEJ) [8] and the Fanconi anemia pathway (FA) [9]. Deficiencies in the repair mechanisms lead to several pathologies, including cancer development, neurological disorders and premature aging [10,11,12]. 

Melanoma originates in pigment-producing melanocytes and is one of the few malignancies with a continuously rising global incidence [13,14]. It is projected to be the fifth and sixth most common cancer in men and women, respectively, and accounts for the vast majority of skin cancer-related deaths [14,15]. Melanoma is one of the most highly mutated cancers, which is in large part attributable to UV light-induced cytidine to thymidine (C>T) transitions [16,17]. When looking at a general landscape of cancer development and progression, genomic instability can be viewed as a metastatic fuel [18,19]. In several types of cancer, genetic instability contributes to the acquisition of a phenotype needed for colonization of distant organs [20,21,22,23,24,25,26] and metastatic progression correlates with an increase in both mutation burden and alteration of genes involved in DNA damage response [22,27,28,29,30,31]. Somewhat surprisingly, despite the immense frequency of genetic alterations in melanoma, it is not associated with somatic defects in DNA repair [32]. On the contrary, melanomas overexpress some of the elements of the DNA repair machinery [33]. Furthermore, the ability of melanoma cells to give rise to distant metastases may rely on a certain level of genetic stability, as evidenced by increased expression of DNA repair associated genes in metastatic tumors, compared to primary lesions [34]. 

Historically, the DNA repair capacity of melanoma cells is considered a potential factor of profound lack of success of systemic treatments. While treatment of melanoma patients has been revolutionized with molecularly targeted therapeutics against the most frequently altered signaling cascade in melanoma—the RAS/RAF/MEK/ERK (MAPK) pathway, the majority of patients relapse within months [35,36,37,38]. Several lines of investigation indicate that treatment of melanoma cells with MAPK inhibitors uncovers DNA damage-associated vulnerabilities in melanoma cells that could be exploited therapeutically [39,40,41,42]. Furthermore, it is becoming increasingly recognized that the efficacy of immunotherapy can be substantially improved by disrupting genome integrity in melanoma cells [43,44,45,46]. In this review, we discuss recent findings concerning the mechanisms that preserve genomic stability of melanoma cells following therapy and present the discourse about the therapeutic potential of targeting DNA repair to improve the survival of melanoma patients.

## 2. Conventional Chemotherapy and Radiotherapy

Genome stability of cancer cells has been a focus of anti-cancer therapy for over a century, as evidenced by the universal use of DNA-damaging chemotherapeutics against virtually all cancer types. Historically, a number of DNA-damaging drugs were used against melanoma, including platinum-based drugs (cisplatin, carboplatin) and alkylating agents (dacarbazine, temozolomide, vincristine, vinblastine, carmustine, fotemustine, paclitaxel) [47]. To date, dacarbazine (DTIC) remains the only FDA-approved chemotherapeutic for the treatment of melanoma [48], although it provides hardly any clinical benefit, as complete responses were observed in less than 5% of patients [49,50]. Similar response rates were obtained with temozolomide (TMZ), an oral analog of DTIC capable of penetrating the blood-brain barrier [51]. DTIC and TMZ are pro-drugs converted to a DNA methylating product via enzymatic conversion in the liver or spontaneous conversion in all tissues, respectively. Methylation of DNA occurs primarily at O^6^ and N^7^ positions on guanine, a reaction which can be directly reversed by methyl guanine methyl transferase (MGMT). While correlation between TMZ resistance and MGMT expression is well established [52,53,54], the results of clinical evaluation of MGMT inhibitors in combination with TMZ were unsatisfactory, which could be explained by the lack of specificity of both drugs and therefore high off-target toxicity that likely precluded administration of effective doses [55]. In general, cytotoxic chemotherapy is largely ineffective against melanoma. Early efforts to increase the efficacy of chemotherapy against melanoma include polytherapy, such as BOLD (bleomycin, vincristine [Oncovin], lomustine, dacarbazine) and CVD (cisplatin, vinblastine, dacarbazine) [56,57], or the addition of immunotherapies such as IFN-α or IL-2, as well as anti-estrogens [58,59,60,61]. Neither attempt, however, has improved objective response, but instead decreased quality of life due to severe side effects of treatment [50]. Despite its profound inefficiency against melanoma, chemotherapy retains a role in palliative care of patients who no longer benefit from other treatment modalities [15]. Similarly, ionizing radiation (IR), with certain exceptions, is rarely used in melanoma patients as a single treatment [15,62,63]. Due to the lack of significant therapeutic response of melanoma patients to ionizing radiation and chemotherapy, the scientific interest in targeting melanoma cell genome integrity has been limited overall. However, recent advances in molecular mechanisms facilitating survival of melanoma cells treated with the current standard-of-care therapeutics reveal potential in disrupting genome stability as a strategy to increase drug efficacy, which will be discussed in detail below.

## 3. Mitogen-Activated Protein Kinase Inhibitors

The present-day melanoma treatment paradigm is in large part dictated by the prevalence of oncogenic activation of the mitogen-activated protein kinase (MAPK) pathway [64]. Because of its fundamental role in melanoma development, the MAPK pathway has been the subject of intensive research, which led to the development of small molecule inhibitors of the BRAF^V600^ mutant protein (vemurafenib, dabrafenib, encorafenib) and the downstream kinases MEK1/2 (trametinib, cobimetinib, binimetinib) [15,35,36,65,66]. The introduction of targeted therapeutics marked a breakthrough in treatment of patients with advanced melanoma: for the first time in decades, a newly developed treatment modality significantly improved melanoma patient survival [65,66]. However, even with combined inhibition of BRAF and MEK, the development of drug resistance is practically inevitable and most patients relapse within a year [35,36]. It is now clear that pharmaceutical inhibition of the MAPK pathway alone will not provide the anticipated cure for melanoma patients and that additional treatment options must be identified.

Large efforts are being directed towards deciphering the molecular determinants of drug resistance. The results of these efforts constantly add to a growing list of mechanisms that underlie the ability of melanoma cells to withstand treatment (recently reviewed in [67]. One of the reasons behind limited therapeutic success is profound intra-tumoral diversity, which results in the coexistence of distinct, phenotypically diverse subpopulations of melanoma cells that exhibit variable sensitivity to treatment [68,69,70,71]. In general, anti-cancer treatments are primarily effective against rapidly proliferating cells, which may lead to the selection of slow-cycling, treatment-resistant cells of a dedifferentiated phenotype that are capable of repopulating the tumor mass [72,73,74,75,76,77,78,79,80]. While the stem-like, tumor-initiating cells comprise a heterogeneous subcategory of cancer cells, it has been observed that their stemness and intrinsic drug resistance depend on a robust DNA damage response and genome stability [81,82,83,84,85,86]. In melanoma, MAPK inhibition has been shown to be less effective against cells with high expression of stem-like markers such as nerve growth factor receptor (NGFR, also known as CD271), JARID1B or AXL [78,87,88]. Interestingly, all three markers partake in the protection of genome integrity.

In addition to its established role in melanoma initiation and metastasis [89,90], NGFR has been shown to regulate genes involved in mitotic stability and DNA repair [91,92,93]. NGFR-responsive genes include Never in Mitosis (NIMA) Related Kinase 2 (NEK2), DNA Topoisomerase II Alpha TOP2A, and RAD51 Associated Protein 1 (RAD51AP1) [92]. NEK2 and TOP2A are nuclear enzymes essential for high-fidelity chromosome separation: NEK2 phosphorylates centrosomal proteins promote splitting of mitotic centrosomes while TOP2A resolves topological states of DNA during replication, transcription and repair [94,95]. RAD51AP1 is a DNA binding protein required for RAD51-mediated homologous repair and FA pathway of DNA damage response [96,97]. Furthermore, gene set enrichment analysis (GSEA) revealed that NGFR-positive melanoma cells were particularly enriched for genes participating in NER and DNA replication [93]. Importantly, knockdown of NGFR increased the frequency of DNA damage in melanoma cells and enhanced their sensitivity to fotemustine [91,92]. Such observations indicate that NGFR not only acts as a phenotypic marker of treatment-resistant cells, but also actively protects melanoma genome integrity, contributing to drug resistance.

Treatment of melanoma cells and tumors with MAPK inhibitors has been shown to induce selection of a preexisting slow-cycling subpopulation of cells with high expression of JARID1B [78,84,98]. JARID1B is a histone demethylase that plays an important role in genome stability: upon double strand DNA break, JARID1B is recruited to the damage site and demethylates H3K4me3, and thereby loosens local chromatin structure and enables Ku70/80 or BRCA1 to assemble at the damage site for NHEJ- or HR-mediated repair, respectively [99]. Pharmacological or genetic inhibition of JARID1B has been shown to induce the accumulation of H3K4me3 at the site of irradiation-induced DNA damage, impair recruitment of DNA repair machinery and compromise repair of the damage [100]. Notably, JARID1B was recently identified as a marker of cross-resistance in melanoma cells treated with BRAF inhibitors and radiation [101].

AXL receptor tyrosine kinase, whose expression is associated with stem-like properties, up-regulation of DNA repair genes and resistance to DNA damaging agents in many cancer types [102,103,104,105,106], has also been reported to be overexpressed in some MAPKi-resistant melanoma cells [88,107]. Furthermore, inhibition of AXL has been shown to aid DNA repair-targeting treatment in several cancers [104,105,108]. In melanoma, AXL inhibition, together with checkpoint kinase 1 and 2 (CHK1/CHK2) silencing, reduced the expression of DNA damage repair proteins and increased apoptosis [109]. CHK1 and CHK2 are central to proper DNA damage response, as they are activated by ataxia telangiectasia and Rad3-related (ATR) protein in response to single-strand break or ataxia-telangiectasia mutated (ATM) kinase, when double strand DNA damage occurs [110]. Although Müller and colleagues reported an inverse correlation between AXL and MITF levels in drug-resistant melanoma [88], drug resistance is not exclusively determined by AXL^high^/MITF^low^ phenotype [67,111]. Melanoma cells may also acquire resistance to MAPK inhibitors through a mechanism dependent on elevated expression of MITF [112,113]. Interestingly, MITF has been observed to promote chemoresistance [114] and genomic and transcriptomic homeostasis [115]. Furthermore, MITF was found to directly regulate the transcription of BRCA1 and FANCA together with a number of genes responsible for DNA repair and replication, including RAD51L3, RAD54, POLM, DNA ligase 1 (LIG1), EME1 and TERT [116,117]. Overall, increased expression of proteins involved, at least in part, in the protection of genome stability in melanoma appears to be a common feature of various mechanisms of drug resistance.

Accumulating evidence suggests that disrupting DNA repair may be an efficient strategy against melanoma. For instance, entinostat, a class I histone deacetylase (HDAC) inhibitor, reduced the expression of RAD51 and FANCD2, which, in turn, sensitized melanoma cells to TMZ and allowed for synthetic lethal targeting of PARP with olaparib [118]. Furthermore, a recent study discovered that dual inhibition of BRAF^V600E^ and MEK with dabrafenib and trametinib potently suppressed the expression of several genes of the homologous repair pathway in a subset of melanoma cell lines, including BRIP1, BRCA2, EME1 and RBBP8 [41]. Maertens and colleagues found that combined suppression of BRAF/MEK together with entinostat resulted in additional downregulation of NHEJ related genes, including XRCC4, XRCC5, XRCC6, PNKP and PARP3, and reduced melanoma cell survival. In search of a predictive biomarker of this drug-sensitivity, the authors found that MGMT expression correlated with sensitivity to combined MAPK and HDAC inhibition [41]. Although MGMT plays a vital role in DNA repair by direct reversion of methyl guanine adducts generated by alkylating agents such as DTIC and TMZ [119], in this setting MGMT expression was deemed a passive biomarker that marked a phenotypic subset of melanomas, without having a functional role in drug sensitivity or resistance [41]. Overall, the study uncovered latent defects in DNA repair of MGMT-expressing melanoma cells exploitable only during MAPK inhibition, as treatment with PARP inhibitor alone was insufficient to exert cytotoxic effects in MGMT-expressing melanomas. Arguably, these findings could have a profound clinical application. Clinical assessment of MGMT promoter methylation, which is currently routinely employed to predict sensitivity of glioblastoma to TMZ [120], could be used in melanoma patients to predict responsiveness to this triple drug combination. Clinical reports indicate that MGMT promoter is methylated, and thus suppressed, in about 21.5–35% of metastatic melanomas [119,121,122,123], which entails that inhibition of BRAF, MEK and HDAC could potentially benefit a large number of melanoma patients. Compatible with findings by Maertens and colleagues, our group has recently reported that vemurafenib and trametinib reduced the expression of BRIP1, BRCA1 and BRCA2 in 4 out of 5 patient-derived BRAF^V600E^ cell lines [42]. In addition, we found that insulin modulated the response of melanoma cells to MAPK inhibition by attenuating drug-induced reduction in the expression of DNA repair genes, deregulation of glutathione homeostasis and increase in DNA damage, which altogether led to substantially reduced cytotoxicity of vemurafenib and trametinib [42]. The role of MAPK-targeted therapeutics in deregulation of DNA repair was also confirmed in other cancer types: BRAF^V600E^ inhibition using vemurafenib was shown to suppress double strand break repair and sensitize thyroid cancer cells to ionizing radiation [124], and MEK inhibition using trametinib induced similar effects in pancreatic cancer cells [125]. Altogether, these reports point to a promising direction in melanoma treatment that could potentially combine MAPK inhibitors with various DNA repair-targeting drugs that have already been FDA-approved.

**Figure 1:**
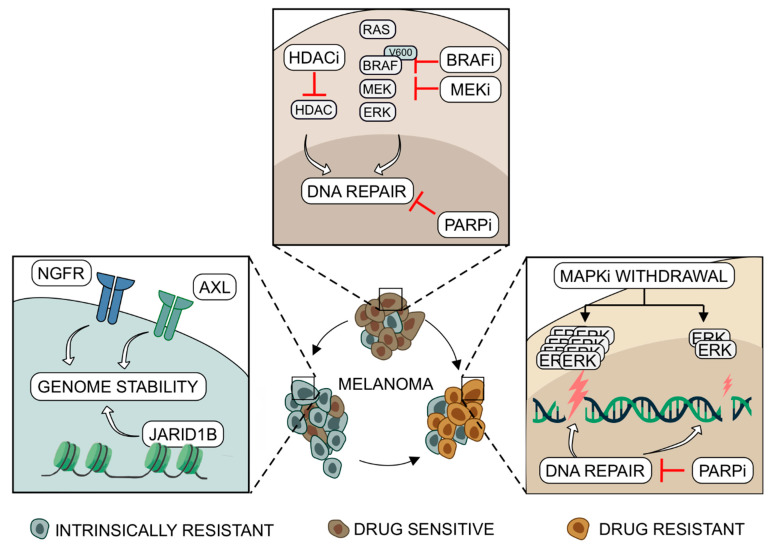
Melanoma cells treated with BRAF and MEK inhibitors depend on efficient DNA repair and genome stability for survival. In drug-naïve melanoma cells, inhibitors of BRAF (BRAFi) and MEK (MEKi) reduce the expression of DNA repair related genes, which can be enhanced by PARP and HDAC inhibitors (PARPi, HDACi) to increase melanoma cell death. Increased expression of NGFR, AXL and JARID1B protects the genome of melanoma cells intrinsically resistant to MAPK inhibitors (MAPKi). Such intrinsically drug-resistant melanoma cells are selected by MAPKi treatment and lead to disease progression. Occurring in parallel, development of acquired drug resistance through reactivation of ERK signaling may result in excessive ERK activity upon immediate drug withdrawal and DNA damage, which can be enhanced by DNA repair inhibitors such as PARP inhibitor (PARPi). Inhibitory effect is marked by red bar-headed arrows.

Once melanoma cells become resistant to MAPK inhibitors, effective therapeutic options are scarce. This prompted intense investigation into specific targeting of MAPKi resistant melanoma cells. One approach to battling therapeutic resistance aims to exploit the apparent drug addiction of melanoma cells: reportedly, melanomas adapted to MAPK inhibition exhibit loss-of-fitness upon drug withdrawal [126,127]. Drug-resistant melanomas are known to circumvent MAPK inhibition and reactivate ERK activity [128,129] and recently, the supra-basal ERK activity induced by drug withdrawal has been reported to be a targetable vulnerability of drug-resistant cells [39]. Depending on the degree of ERK hyperactivation, it caused cell cycle deceleration or DNA damage and parthanatos-related cell death [39]. Pharmacological suppression of DNA repair with ATM and PARP inhibitors augmented the drug addiction phenomenon, leading to caspase-dependent cell death in slow-cycling cells with innately weak phospho-ERK rebound or accelerated parthanatos-related death in cells with excessive ERK activity [39]. These findings suggest there is a therapeutic window for targeting DNA repair in patients who progressed on MAPK inhibitors and no longer receive treatment, regardless of the initial mechanisms underlying drug resistance (Figure 1). Another approach against drug-resistant cancer cells is to identify and target potential vulnerabilities that developed in parallel with drug resistance. For instance, melanoma cells resistant to MAPK inhibitors exhibited reduced expression of TAp73alfa, which binds to p53-responsive genes and modulates DNA repair and genomic stability [40]. Downregulated levels of TAp73alfa resulted in reduced NER capacity in melanoma cells and enhanced susceptibility to DNA cross-linking agents as consequences of accumulating DNA damage [40]. Moreover, it was found that drug-resistant melanoma cells rely on RAD51 expression for survival and can be successfully targeted with Rad51 inhibitors [33]. Rad51 is an indispensable protein of the homology-directed DNA repair pathway and is critical for the maintenance of genomic stability [7]. Its expression in melanoma cells is in part regulated by Elk1 as an outlet of MAPK signaling [33,130]. Inhibition of RAD51 led to the accumulation of DNA damage and enhanced drug efficacy in drug-naïve, vemurafenib-resistant and vemurafenib and trametinib double-resistant melanoma cells, as well as in a xenograft of melanoma cells isolated from a patient with developed resistance to vemurafenib [33]. Notably, Rad51 inhibitors showed no apparent toxicity in mice, which warrants further research into clinical application of Rad51 inhibitors in the treatment of cancers, including melanoma [33]. Drug-resistant melanoma cells have also been shown to lose phosphatase and tensin homolog (PTEN) expression to maintain the pro-survival signaling of the PI3K/AKT pathway [131,132]. Interestingly, cells deficient in PTEN exhibited compromised chromosomal stability, DNA replication and NHEJ repair [133,134,135,136], which makes PTEN-deficient melanomas potential candidates for synthetic lethal targeting of the redundant DNA repair proteins. Enhanced lethality in PTEN-null melanoma tumors has been reported following PARP, RAD51 and ATR inhibition [137,138]. Notably, loss of PTEN expression is frequently observed in melanomas irrespective of MAPKi resistance. Although genetic alterations in PTEN are found in only about 15% of metastatic melanomas [139], epigenetic silencing of PTEN is common at different stages of the disease [140,141].

Lastly, several groups have proposed to target DNA repair proteins in melanoma cells independently of MAPK inhibitors. Santamaria and colleagues reported that melanoma cells depend on the expression of lysyl oxidase-like 3 (LOXL3) for survival [142]. Gain- and loss-of-function studies revealed that LOXL3 contributed to melanomagenesis, protected genome integrity of melanoma cells and physically interacted with other proteins involved in genome integrity maintenance, such as BRCA2 and DNA mismatch repair protein MSH2. Loss of LOXL3 resulted in aberrant DNA damage response, characterized by inefficient ATR activation and a substantial decrease in protein levels of BRCA1, BRCA2, MSH2 and Rad51, altogether leading to the accumulation of double-strand DNA breaks and aberrant mitosis [142]. Another group has reported that disruption of DNA repair and epigenetic state by knockdown of a BER enzyme thymine DNA glycosylase (TDG) may be a potent strategy against melanoma, and furthermore, the group also evaluated candidate inhibitors of TDG for the treatment of melanoma [143]. Finally, CC-115, a dual inhibitor of mTOR and DNA-PK, has been shown to be cytotoxic and radiosensitizing in melanoma cells and is currently under clinical evaluation [144].

## 4. Immunotherapy

**Figure 2:**
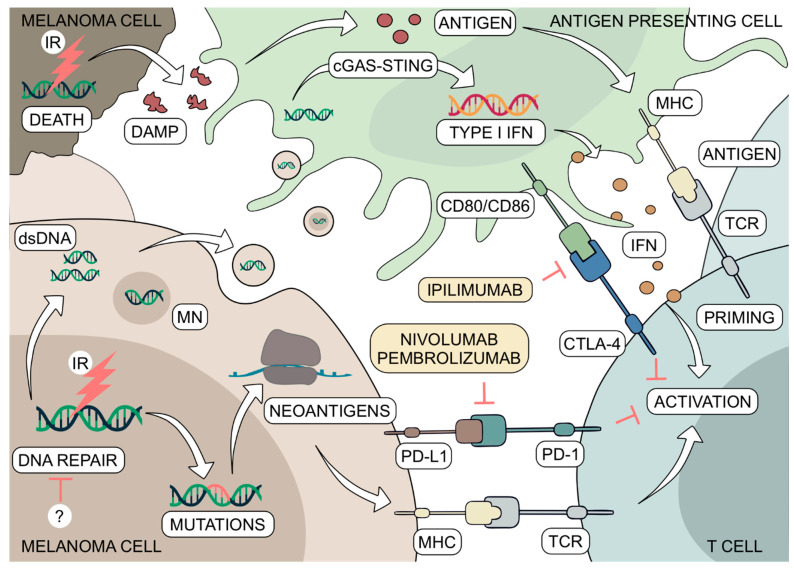
Mechanisms of irradiation-induced stimulation of immune system. Ionizing radiation (IR) leads to DNA lesions that may result in mutant proteins and neoantigen presentation via MHC molecule for T cells to recognize. IR-induced DNA damage may cause cytosolic DNA or micronuclei (MN) accumulation and secretion, which is taken up by antigen presenting cells, and leads to cGAS-STING pathway-mediated type I interferon production and secretion. Finally, severe damage by IR can cause cell death and release of DAMP molecules, which are processed by antigen presenting cells and presented to T cells leading to T cell priming. Immunoinhibitory interaction of PD-L1 expressed by melanoma cells with PD-1 present on T cells can be targeted with nivolumab and pembrolizumab, while CD80/CD86 and CTLA-4 interaction between antigen presenting cell and T cell can be inhibited by ipilimumab. Inhibitors of IR-induced DNA repair are likely to augment the genome destabilizing immunostimulatory effect of IR. Inhibitory effect is marked by red bar-headed arrows.

As with MAPK pathway inhibitors, the past decade brought groundbreaking advances in immunotherapy for melanoma patients. The therapeutic paradigm has shifted from a relatively ineffective cytokine-based treatment to antibody-mediated immune checkpoint inhibition that provides exceptionally durable responses [145]. Currently used immunotherapeutics target cytotoxic T-lymphocyte-associated antigen 4 (CTLA-4) or programmed cell death receptor 1 (PD-1) and prevent them from binding to CD80/CD86 or PD-L1, respectively (Figure 2). These particular ligand-receptor interactions constrain lymphocyte activation in a non-redundant manner [146,147]. Nivolumab and pembrolizumab, which are both anti-PD-1 antibodies, demonstrate superior clinical benefit over CTLA-4 blocking ipilimumab and remain the standard of care for melanoma patients whose tumors do not harbor BRAF^V600^ mutations [148,149]. Although immunotherapy provides unprecedentedly durable benefit in some patients, a substantial percentage of patients relapse within 2 years of treatment and approximately half of patients do not achieve significant therapeutic response at all [150,151,152,153]. Therefore, the ultimate challenge of immunotherapy is to sensitize drug-refractory melanomas that evade immune recognition and prevent relapse in immunotherapy-responsive individuals.

Molecular determinants of effective immunosurveillance include various interdependent host and tumor cell factors, which have been described in greater detail elsewhere [154,155,156,157]. In short, anti-tumor activity of the immune system depends on the presence of tumor-specific antigens on the melanoma cell surface, immune infiltration of the tumor and activation of the immune cell response [158]. The expression and altered repertoire of antigens is driven by large quantities of somatic mutations, which in melanoma, on average, is greater than in many other tumors [159,160]. The mutation burden, however, varies between patients, which renders some melanoma tumors less immunogenic [160,161,162,163]. Furthermore, for neoantigens to generate immunological response, the cell needs to properly process and present neoepitopes by the major histocompatibility complex class I (MHC class I), which is often downregulated in melanomas [164,165]. A critical prerequisite for successful immunotherapy is the presence of immune cells inside the tumor [166,167]. Finally, the outcome of melanoma and immune cell encounters depends on sufficient activation of the immune cells present in the tumor microenvironment, which melanoma cells prohibit by upregulating the expression of programmed death-ligand 1 (PD-L1) [168].

Considering that all the aforementioned factors are indispensable for full anti-tumor immunological response, it has been increasingly recognized that DNA damage, particularly when induced by ionizing radiation, may serve as an immunotherapy-augmenting treatment [169]. Ionizing radiation induces a variety of DNA lesions that have the potential to be cytotoxic or mutagenic, both of which can stimulate an innate and adaptive immunological response [170,171] (Figure 2). Damaged DNA increases the likelihood of neoantigen formation, enhances the expression of MHC class I loaded with unique, irradiation-induced peptides and as a result, alerts the immune system of melanoma cells [172,173]. Furthermore, irradiation can lead to an immunogenic cell death, associated with the release of damage-associated molecular pattern (DAMP) molecules which boost immunological defense [174]. Radiation can induce the accumulation of cytosolic DNA, either in the form of micronuclei generated from chromosomal aberrations or small double-stranded DNA fragments that leak through a ruptured nuclear envelope [175]. Cytosolic DNA can then trigger inflammatory signaling in cancer cells or can be released and sensed by immune cells within the tumor microenvironment. It has been shown that ionizing radiation enhances melanoma-derived DNA uptake by antigen presenting cells (APC) and promotes anti-tumor immunity [176,177]. Mechanistically, the presence of cytosolic, tumor-derived DNA in immune cells is recognized by cyclic GMP-AMP synthase (cGAS) which activates stimulator of interferon (IFN) genes (STING), leading to IFN-beta production and CD8+ T cell priming [178]. Importantly, anti-tumor efficacy of radiotherapy has been shown to substantially rely on host cell STING signaling, rather than direct cytotoxicity of ionizing radiation in melanoma cells [179].

With all the immunostimulatory effects of DNA damage, it is worth emphasizing that radiotherapy as a single agent is not effective against melanoma, at least not in terms of cure [180]. As with chemotherapy, the efficacy of radiotherapy relies on the DNA repair capacity of targeted tumor cells and is substantially more effective in cancers with deficiencies in DNA repair [181]. Nonetheless, radiation temporarily shrinks melanoma tumors, which denotes some degree of cancer cell death, and this in turn can be leveraged with immunotherapy [48]. Most importantly, radiotherapy has been shown to increase tumor immune infiltration, which is also demonstrated by increased expression of PD-L1 on the surface of melanoma cells as an adaptive mechanism against immune cell mobilization inside the tumor mass [182,183]. Given that the preexisting lymphocyte infiltration is crucial for immunotherapy efficacy [158], this otherwise drug-efficacy-lowering PD-L1 expression ultimately results in a substantially more effective tumor cell killing after anti-PD-1 treatment. Furthermore, radiotherapy combined with immunotherapeutics has been shown to generate tumor antigen-specific memory T-cell responses and limit T-cell exhaustion [182,184]. Radiation can also induce an immune-mediated phenomenon called the abscopal effect, in which irradiation of one tumor leads to the regression of a non-irradiated tumor at a distance from the irradiated site [185]. Mechanistically, this effect is based on DNA damage-induced mobilization of systemic immunosurveillance, particularly mediated by cGAS-STING signaling [186]. Several clinical reports have demonstrated the abscopal effect in melanoma patients who received radiotherapy prior to treatment with anti-CLTA-4 [186,187,188] and anti-PD-1 antibodies [189,190,191]. However, the sequence and timing of treatment is believed to be a crucial determinant of the efficacy of this therapeutic strategy. Dovedi and colleagues found that irradiation-induced CD8+ T cell infiltration sharply decreased after 7 days post radiation, which abrogated the synergistic effect of radiotherapy and PD-1/PD-L1 blockade [182]. Another study found that inflammatory signaling after radiotherapy-induced DNA damage was dependent on mitotic progression, which generated micronuclei and stimulated cGAS-STING signaling. Both inhibiting mitosis as well as the loss of STING signaling impaired interferon signaling and the loss of STING signaling limited the abscopal effect of radiation [186]. Hence, allowing time for tumor cells to divide was necessary for the immunostimulatory effects. Most clinical reports have postulated that combined treatment provides superior benefit when it is not substantially stretched over time [192]. Concurrent administration of anti-CTLA-4 or anti-PD-1 treatment in patients who underwent stereotactic radiosurgery of metastatic lesions in the brain significantly improved median tumor reduction comparing to non-concurrent immunotherapy [193]. Furthermore, overall survival of patients who received ipilimumab within 14 days of radiotherapy was significantly greater than in patients who underwent radiotherapy 4 months prior to immunotherapy [194]. Altogether, substantial benefit of combining immunotherapy with radiation for melanoma patients has been proven [43,44,45,46]. On the other hand, a retrospective study comparing immunotherapy with or without preceding radiotherapy reported no evidence of favorable outcome of pretreatment radiotherapy. However, the study was not set up to determine the time-from-radiotherapy impact on the efficacy of immunotherapy, and included stage IV patients who underwent radiotherapy at a non-specified time prior to immunotherapy administration [195]. This study, however, is a good example of the need of strict control of treatment schedule.

Overall, as evidenced by the use of radiotherapy, DNA damage exposes tumor cells to the immune system and stimulates T cell activation. This is further illustrated by the association between defects in DNA repair and the efficacy of immunotherapy [196,197], particularly in tumors with mutations in MMR genes, in which exceptional responses to pembrolizumab have been observed [198,199,200]. Notably, genomic landscape of triple wild-type melanomas (lacking mutations in RAS, RAF and NF1 genes), which account for 5–10% of melanomas, was recently characterized as being enriched for mutations in DNA repair-related genes [201]. Although overall rare in melanoma, mutations in DNA repair-related genes were also associated with enhanced response to immunotherapy. For instance, an unprecedented overall survival rate exceeding 7 years was reported for a patient with metastatic melanoma lacking the expression of MMR gene MSH6 [202], while in a preclinical melanoma model, knockdown of MSH2 in poorly immunogenic tumors markedly improved the response to anti-PD-1 immunotherapy [203]. Furthermore, presumed loss-of-function mutations in BRCA2 were found to be significantly more frequent in anti-PD-1 treatment responsive tumors [152]. It logically follows that targeting DNA repair together with irradiation of melanoma tumors could amplify the immunogenic DNA damage. Combination of radiotherapy with AsiDNA, a DNA repair trapping molecule that hijacks HR and NHEJ proteins, has been well tolerated in mice [204] and encouraged phase I clinical evaluation of this treatment modality [205]. Tourneau and colleagues reported complete responses in 30% of patients and speculated that immunogenicity-induced abscopal effect contributed to the systemic response to locally injected treatment [205]. Simultaneous targeting of DNA repair with immunotherapy has also been successful in BRCA-deficient cancers, which serves as an example of how decreased genome stability synergizes with immune checkpoint blockade [206,207]. Granted, cells deficient in HR due to mutations in BRCA genes are 100–1000-fold more sensitive to pharmaceutical inhibition of PARP; however, a study analyzing PARP inhibition in combination with AsiDNA in HR-proficient tumors showed that this drug combination can recapitulate synthetic lethality in tumors, regardless of their HR mutation status [208].

## 5. Conclusions and Future Directions

MAPK pathway antagonists and immune checkpoint inhibitors will likely remain the cornerstone of melanoma treatment. However, it is clear that additional targets need to be identified to maximize treatment efficacy, eliminate subpopulations of melanoma cells that are intrinsically resistant to treatment and prohibit the development of drug resistance. A growing body of evidence supports the hypothesis that melanoma cells depend on a certain level of genome stability when undergoing drug treatment and that inducing DNA damage may augment the efficacy of MAPK inhibition and immune checkpoint blockade. While anti-tumor effects of BRAF and MEK inhibitors could be enhanced with drugs targeting proteins involved in DNA repair, immunotherapy may draw benefit from directly interfering with the DNA integrity. Several clinical trials are under way to evaluate the safety and efficacy of treatment regimens that combine MAPK or immune checkpoint inhibitors with chemotherapy and radiotherapy or drugs that potentially destabilize genome integrity (Table 1). Such drugs include inhibitors of histone deacetylases (HBI-8000, 4SC-202 or entinostat), PARP (olaparib, niraparib and talazoparib) and ATR (ceralasertib/AZD6738). However, along with measuring response rates to treatment, still many clinical trials are prominently focused on assessing dosage limited toxicities and incidences of adverse effects. Nonetheless, the outcome of these studies may pave the way for research into targeting melanoma genome stability in order to enhance the efficacy of current first-line treatment options. The majority of ongoing phase 2 and 3 clinical trials test the combination of immunotherapy with genome-targeting treatments (Table 1). Notably, these studies are not powered to establish the optimal treatment schedule, which may be a crucial determinant of the treatment outcome. Therefore, it would be beneficial to set out clinical evaluation aimed specifically to elucidate the best radiation and immunotherapy timeline. On the other hand, only a few of the current clinical trials examine the therapeutic potential of MAPK inhibitors combined with radiation, one evaluates combination of MAPKi with an AXL inhibitor and none involve drugs targeting proteins of the DNA repair pathways or histone deacetylases (Table 1). This is likely because preclinical data that points to an exploitable dependency on genome stability in melanoma cells treated with MAPK inhibitors is fairly recent and not yet extensively explored. In summary, combining current anti-melanoma drugs with therapeutic agents that destabilize the melanoma cell genome and interfere with DNA repair offers great promise in melanoma treatment and further studies on both preclinical and clinical levels are warranted.

## 6. Methodology

To comprehensively cover studies relevant to the investigated topic, a literature search was conducted using Pubmed, Scopus and Web of Science, based on the following keywords, alone or in combination: melanoma, DNA damage, DNA repair, targeted therapy, immunotherapy, chemotherapy, radiotherapy, genome stability. Preference was given to papers published within the past 10 years. All information presented in this review has been thoroughly examined and discussed between the authors.

A summary of clinical trials presented in Table 1 was prepared based on clinicaltrials.gov (accessed on 23 March 2021) database [209] using “melanoma” as the keyword and filtered by: ”recruiting”, ”not yet recruiting” and ”active, not recruiting” to focus only on ongoing studies. The initial list yielded 923 hits. The search was narrowed down to clinical trials involving skin cutaneous melanoma and excluded phase 1 clinical trials to focus on studies measuring the outcome of treatment instead of only the appropriate dosage of treatment. Finally, studies that involved a combination of immune checkpoint or MAPK inhibitors with genotoxic agents as well as studies evaluating drugs targeting DNA repair alone or in combination with immune checkpoint or MAPK inhibitors were selected.

## Figures and Tables

**Table 1:** Ongoing clinical trials in the treatment of melanoma that combine MAPK inhibitors or immune checkpoint inhibitors with chemotherapy, radiotherapy or drugs targeting proteins of relevance to genome stability.

Identifier	Phase	Enrollment	Primary Outcome Measures	Treatment Regimen Description
NCT01676649 ^1^	2	30	adverse events	ipilimumab + carboplatin + paclitaxel
NCT02097732 ^1^	2	4	LCR	SRS + ipilimumab
NCT0239287 ^1^	1/2	10	adverse events and radiotherapy associated toxicities	radiotherapy + dabrafenib + trametinib
NCT02617849 ^1^	2	30	ORR	carboplatin + paclitaxel + pembrolizumab
NCT02718066 ^2^	1/2	118	RP2D	HBI-8000 (HDACi) + nivolumab
NCT0281602 ^2^	2	71	ORR	azacytidine(cytidine analog) + pembrolizumab
NCT02872259 ^2^	1/2	92	ORR	BGB324 (AXL inhibitor) + pembrolizumab BGB324 + dabrafenib + trametinib
NCT02974803 ^1^	2	6	intracranial OR	SRS + dabrafenib + trametinib
NCT02978404 ^1^	2	26	intracranial PFS	nivolumab + radiosurgery
NCT02988817 ^2^	1/2	374	DLTs, adverse events	enapotamab vedotin (HuMax-AXL-ADC)
NCT03050060 ^2^	2	129	ORR	nelfinavir mesylate + pembrolizumab, nivolumab, or atezolizumab + hypofractionated radiation therapy
NCT03278665 ^2^	1/2	40	IAE	4SC-202 (HDACi) + pembrolizumab
NCT03340129 ^2^	2	218	intracranial response to immunotherapy	nivolumab + ipilimumab + SRS
NCT03425279 ^2^	1/2	120	DLTs, MTD, ORR	CAB-AXL-ADC (anti-AXL antibody drug conjugate)
NCT03430947 ^2^	2	32	ORR in brain	radiosurgery + vemurafenib + cobimetinib
NCT03448666 ^3^	2	53	ORR	electrochemotherapy + pembrolizumab
NCT03474497 ^2^	1/2	45	ARR	pembrolizumab + IL-2 + hypofractionated radiotherapy.
NCT03511391 ^1^	2	99	PFS	nivolumab or pembrolizumab or atezolizumab + SBRT
NCT03646617 ^2^	2	70	number of adverse events	ipilumumab + nivolumab + HFRT
NCT03693014 ^2^	2	60	ORR	SBRT + ipilimumab, nivolumab, pembrolizumab or atezolizumab
NCT03765229 ^2^	2	14	ORR, PFS	entinostat (HDACi) + pembrolizumab
NCT03780608 ^1^	2	61	ORR	ceralasertib (ATR inhibitor) + durvalumab (PD-1/PD-L1 inhibitor)
NCT03898908 ^2^	2	38	intracranial ORR	encorafenib + binimetinib + radiation
NCT03925350 ^2^	2	41	ORR	niraparib (PARPi)
NCT03958383 ^2^	1/2	61	IAE, MTD, MAD	radiation + nivolumab + ipilimumab + hu14.18-IL2
NCT04017897 ^2^	2	52	ORR	pembrolizumab or nivolumab + radiotherapy
NCT04042506 ^2^	2	15	safety of SBRT	SBRT + nivolumab
NCT04074096 ^3^	2	150	intracranial PFS	SRS + encorafenib + binimetinib
NCT04133948 ^2^	1/2	45	safety of patients	domatinostat (HDACi) + nivolumab + ipilimumab
NCT04187833 ^2^	2	37	best overall response (CR + PR)	nivolumab + talazoparib (PARPi)
NCT04225390 ^2^	2	38	CR, PR, SD or PD	DTIC + re-exposure to immunotherapy
NCT04594187 ^2^	3	168	time to regional nodal recurrence	nodal radiation therapy + immunotherapy
NCT04620603 ^3^	1/2	15	tumor response	low dose rate brachytherapy + nivolumab
NCT04633902 ^3^	2	41	ORR	olaparib (PARPi) + pembrolizumab
NCT04793737 ^2^	N/A	27	ORR	precision radiation in patients on PD-1 inhibitor treatment that have tumor progression

^1^ active, not recruiting; ^2^ recruiting, ^3^ not yet recruiting; ACT, adoptive cell therapy; ARR, abscopal response rate; CR, complete response; DLTs, dosage-limiting toxicities; EFS, event-free survival; HFRT, hypofractionated radiotherapy; IAE, incidence of adverse effects; LCR, local control rate; MAD, maximum administered dose, MTD, maximum tolerated dose, N/A, not applicable; OR, objective/overall response; ORR, objective response rate; PD, progressive disease; PFS, progression-free survival; PR, partial response; RP2D, recommended phase II dose; SBRT, stereotactic body radiation therapy; SD, stable disease; SRS, stereotactic radiotherapy.

## Data Availability

Not applicable.
